# Outcome prediction abilities of basketball players shooting free throws

**DOI:** 10.1371/journal.pone.0330545

**Published:** 2025-08-22

**Authors:** Lea Elisabeth Junge-Bornholt, Fabian Dominik Wachsmann, Heiko Maurer, Mathias Hegele, Hermann Müller, Lisa Katharina Maurer

**Affiliations:** 1 Neuromotor Behavior Laboratory, Department of Psychology and Sport Science, Justus Liebig University, Giessen, Germany; 2 Center of Mind, Brain and Behavior, Universities of Marburg, Giessen and Darmstadt, Germany; 3 Experimental Psychology, Justus Liebig University, Giessen, Germany; Sheffield Hallam University, UNITED KINGDOM OF GREAT BRITAIN AND NORTHERN IRELAND

## Abstract

Skilled athletes, such as basketball players, seem to anticipate the outcome of their actions, likely due to well-developed internal models that enhance predictive accuracy. This study explores whether experienced basketball players can verbally predict their free throw outcomes above chance, examining the role of expertise and potential biases in prediction accuracy. Therefore, 19 experienced basketball players performed 500 free throws in a laboratory setting. Within 2.5 seconds of visual and acoustic occlusion after ball release, they had to predict the result of every second trial as either a hit or a miss. Individual prediction accuracies above chance were calculated, and the hit bias was quantified by a signal detection theory measure (criterion *c*). Speech characteristics (latency and amplitude) were used as an additional proxy to investigate the prediction process. It was hypothesized that experienced players would make faster predictions of successful shots and would articulate these with greater volume compared to unsuccessful ones, reflecting the processing of available information about their throw execution and heightened response bias toward success. Results showed that participants were able to significantly predict their free throw outcomes above chance level. An earlier described hit bias could be replicated and was further observed as a louder and faster articulation of hits than misses. Overall, natural motor experts, in this case basketball players, seem to access their internal models and use the gathered information to predict the outcome of their free throws. However, they show a bias to predict hits, which is also manifested in the speech characteristics of latency and amplitude.

## Introduction

To act in and react to an ever-changing environment, the human central nervous system has to integrate interoceptive and exteroceptive information [1]. Internal models facilitate this process by estimating future consequences to optimize motor behavior. Two primary types of internal models exist: inverse models determine the necessary motor commands to achieve a desired sensory state [2], while forward models predict the sensory consequences of motor commands [3]. It has been shown that predicting sensory consequences enables real-time adjustments of movements, thereby reducing reliance on constant sensory feedback and enabling smoother, faster actions in dynamic environments [4]. Hence, predictive models enhance efficiency by minimizing surprise and optimizing behavioral responses. Forward models are constantly updated by comparing predicted and actual sensory feedback, making them essential for motor learning and adaptation [5]. With the increasing complexity of actions, prediction difficulty also increases, requiring greater effort to develop a reliable forward model [6]. From this, it can be inferred that practice should strengthen these models by improving the coupling between motor commands and sensory consequences [7]. Thus, people with extensive experience in certain motor tasks (i.e., motor experts) should have acquired highly reliable internal models. This should enable them to access and use the predictive information explicitly in planning appropriate responses to current situations (e.g., rebound after a basketball shot). The definition of a “motor expert” varies widely among studies (for a review [8]). We will use the term “motor expert” to refer to athletes with many years of experience in a motor skill and who regularly train and perform competitively.

Research has used throwing movements to study the accessibility of internal predictions because of the natural delay between movement and outcome in goal-oriented throws (e.g., [9–11]). In a virtual throwing task, it has been shown that already non-expert but trained participants could verbally predict the outcomes of their throws with an accuracy above individual chance levels. However, the individual prediction accuracies largely varied between participants and were related to their task performance [12]. While controlled laboratory tasks have been invaluable for testing hypotheses about predictive processing, there is a growing need to extend these investigations to natural, real-world tasks involving complex movements with many degrees of freedom.

Studies on natural basketball experts examined outcome predictions of observed motor actions. For instance, a study on basketball free throws showed that expert players outperformed non-players in prediction performance [10]. Further, this superiority was primarily based on the player’s own playing experience [10,13]. Natural motor experts obviously can use visual information better than non-experts to predict movement outcomes. Two other studies focused on outcome predictions of self-executed basketball free throws. While visual feedback was manually omitted using liquid crystal goggles, basketball experts had to categorize their shots as either made or missed. Executing players were superior in the prediction of their throws compared to the simultaneous prediction by observing players [9], and players with more experience outperformed less experienced ones in hit trials [14]. Thus, it seems that expert players are indeed able to use information from their internal prediction processes to discriminate hit from miss trials. Interestingly, both studies observed a response bias of experts toward predicting successful shots, regardless of the actual result.

The present study builds on this previous work on the accessibility of internal predictions about the outcome of executed actions within a natural context. More specifically, it was examined whether experienced basketball players can verbally predict the outcomes of their own free throws above chance level, how a possible response bias may influence their verbal reports, and what the cognitive mechanisms underlying these predictions are. Further, it was tested whether the prediction ability and the response bias increase with shooting performance, respectively.

While movement outcome prediction has been well documented, how internal motor predictions translate into verbal binary outcome judgments remains unclear. These motor predictions likely operate within lower levels of the motor control system, guiding movement control and learning. In the following, a framework is proposed for how this predictive process can be conceived. In doing so, the acoustic features of the verbal utterances were linked to certain states in the processing stream as proxies for details of inner processing.

When people are requested to verbally predict the future outcome of an action they just performed, they must advance from an initially uninformed state to a posterior informed state. Before the movement, only nonspecific information on how well the movement is generally executed is available. Relying solely on this prior information, participants should always say “hit” if their average hit rate exceeded 50%, and, respectively, always say “miss” if the average hit rate was below 50%. This strategy would lead to the highest prediction accuracy if no information on the actual throw is considered in the prediction process. However, the studies mentioned above have shown that people can exceed this baseline for prediction accuracy. The only way this is possible is by incorporating additional real-time motor and sensory information into the formation of their belief as to whether that particular throw will hit. That is, people may rely on their internal models and include knowledge about the commands sent to the effectors, or they may additionally integrate information from sensors that monitor the flow of events during movement execution. Yet, it should be noted that any single sensed deviation from an intended progression of the movement is insufficient to justify the prediction of a miss since most throwing tasks are redundant (e.g., [15]). This means that multiple solutions exist, e.g., a deviation in the throwing angle might be compensated by an opposite deviation in release velocity. Therefore, all available information must be integrated and run through a prediction model to infer whether a given sensed situation will end in a hit or miss. Due to the necessity of such computations, it takes time to integrate incoming information from different sensors and derive a prediction regarding the expected outcome [16]. Furthermore, the various sources of information become available at different moments in time: before, during, or shortly after movement execution [17]. Consequently, it is proposed that internal predictions are generated continuously rather than at a single moment and that prediction accuracy increases as more information is processed. When participants reach a certain confidence threshold, they should be convinced of this result and report the respective status of their inner belief. Ambiguous trials, where execution deviates from optimal patterns, may require more processing time and may result in higher incorrect predictions.

The time window required to integrate the available information and generate a prediction can be quantified as the response latency [18]. Given the hit bias observed in motor experts (e.g., [9,19]), it can be assumed that their inner belief requires less information and time to reflect a hit than a miss, leading to faster articulations of successful outcomes. A more pronounced bias is expected to further enhance this difference between the two response options. Additionally, higher response latencies in incorrect predictions were expected due to prolonged processing of ambiguous information. Considering the hit bias of athletes as well as a generally increased amplitude in the articulation of positive emotions and states of high arousal [20], louder expressions of hits than misses were expected from experienced basketball players. These assumptions would fit the results from previous work, where earlier and louder verbal reports were found when participants predicted successful outcomes [12].

## Materials and methods

### Participants

Nineteen experienced basketball players (6 female, 13 male) with an average of 11.2 and a standard deviation (SD) of 4.9 years of basketball training participated in the study. Participants’ mean age was 23.7 (SD = 4.8) years (range: 14−37 years). The sample size was determined based on a similar study on outcome prediction in a virtual throwing task. This study reported an effect size of *d* = 0.63 for the measure of prediction accuracy above the individual chance level, as determined by a one-sample t-test [12]. Using this effect size, a power analysis (with *α* = 0.05) indicated that a sample size of 18 participants would achieve a power of 0.82. Players had to be part of a competitive basketball team and train at least twice a week. As the data was collected during the COVID-19 pandemic, this criterion was considered to be met if they had fulfilled it at least before the pandemic. The sample included athletes from the second division of the German Basketball League, as well as from higher regional teams. Based on their league and years of training, they match the description of previous studies examining “experienced”, “intermediate”, or “collegiate” basketball players [14,21,22]. While recruiting from basketball teams with comparable skills and age might reduce the variance in the dataset, it would also limit the generalizability of the study. The participants received course credit or 8 € per hour as compensation. The study protocol was conducted in accordance with the “World Medical Association Declaration of Helsinki” ([23] 2013, except for §35, pre-registration), and approved by the Ethical Review Board of the Justus Liebig University, Giessen (2017−0033). Recruitment and data collection took place from 21 June 2021–28 August 2021.

### Apparatus

A standard basketball board and ring at the official height (National Basketball Association) were set up for the data acquisition, and the free-throw line was marked with tape 4.225 m away from the center of the ring. Standard National Basketball Association basketballs of sizes 7 and 6 were used for men and women, respectively. Participants wore their personal basketball gear and shoes, which they used for regular indoor training, and were allowed to perform individual rituals.

Movements of the players and ball trajectories were recorded with a passive marker-based optoelectrical camera system (Vicon Motion Systems, Oxford, UK). 28 Vicon Vantage V5 cameras were optimized for a volume of 5 m x 2 m x 4.5 m (length, width, height) and sampled with 240 Hz. Before starting the data acquisition of a new participant, the cameras were calibrated using an Active Wand V2 according to the manual of the Vicon Nexus 2.12 software, and capturing at least 3’000 frames at a sampling rate of 240 Hz. The calibration was accepted when the residual errors for each camera were below 1 mm, based on the calibration feedback provided by the Vicon Nexus software.

Four 14 mm Soft X-Base reflective markers were placed on the basketball surface at the left and right medial-lateral axis and approximately 10 and 15 cm from the right axis marker in two dimensions to form a triangle. A 14 mm Pearl Hard Base marker was attached near the tip of the middle finger of the player’s throwing hand, positioned over the fingernail region. This marker was used to calculate the ball-fingertip distance in real-time and to identify the moment of release (see below for detailed descriptions). Three additional markers were placed at the player’s hand and wrist: one on the lateral side of the wrist at the *processus styloideus ulnae*, another on the dorsal side of the wrist approximately halfway between the *processus styloideus ulnae* and the *processus styloideus radii,* and the third on the back of the hand just proximal to the third metacarpophalangeal joint. These markers were not used for any calculations. Rather, they were necessary to enable the Vicon Nexus software for real-time labeling, which relies on the configuration of multiple markers. The marker set was developed as a result of previous pilot work, in which a stable labeling of the four markers was confirmed, as well as the correct occlusion timing of the liquid crystal goggles (see below for detailed information).

To ensure that the verbalized throwing results provided by participants were based on predictions, visual and auditory feedback after ball release was deprived. To this end, participants wore liquid crystal PLATO goggles (Translucent Technologies Inc., Toronto, Canada) occluding the sight for 2.5 s after ball release. The goggles were closed automatically based on the kinematic information of the ball provided by the Vicon system. Based on pilot research where kinematic data and high-speed video (120 Hz) were captured synchronously, an increase in the distance between the fingertip and the ball center (calculated as the midpoint between the two pole markers) was identified as the most sensitive criterion for determining the moment when the ball loses contact with the hand. A threshold of 0.003 m from one frame to the next was found to be sufficiently robust to detect this increase. However, video recordings revealed a temporal delay of approximately 40 ms in closing the liquid crystal goggles when applying this criterion in online recordings. This delay is attributed to various processing steps in the experimental setup: (1) 3D reconstruction and labeling in Vicon Nexus. (2) Kinematic data transmission. (3) Data Processing in experimental software. (4) PLATO goggle activation.

To compensate for these delays, an alternative criterion was developed that allowed for anticipating the ball release and initiating the closing of the liquid crystal goggles before actual loss of hand contact. Ten throws from the warm-up trials were analyzed (see Design and Procedure) to determine the average ball height achieved 40 ms before ball release for each participant. This individual ball height criterion was used to initiate the closing of the PLATO goggles during the experimental trials. To prevent false detections of ball release, additional criteria must be met to confirm ball release (a ball speed greater than 5 m/s and a duration of more than 0.5 s since ball contact).

To withdraw auditory feedback, participants wore Bluetooth Soundcore Life Note in-ear headphones (Anker Innovations Ltd., Shenzhen, China) playing white noise as soon as the trial started, as well as soundproof Peltor Optime 1 on-ear headphones (3M Company Corp., Saint Paul, Minnesota, United States) comparable to previous research [9,14]. Participants’ verbal predictions were captured by an MX153 Earset Microphone and the GLXD14 Digital Wireless Guitar System (Shure Incorporated Corp., Niles, Illinois, United States), using a sampling frequency of 100 kHz and a  + 20 dB gain. Speech data was collected by a BNC-2110 connector block and a PCIe-6321 Multifunction I/O Device (National Instruments AG, Austin, Texas, United States). All attachments to the participants were wireless to enable naturalistic and unrestricted movements. A lightweight harness was used to fixate the transmitter and receiver boxes of the goggles and the microphone.

Data Acquisition Toolbox Version 4.3 was used with MATLAB (The MathWorks Inc., Natick, Massachusetts, United States) version R2021a to control the experiment. In addition, the Datastream SDK Version 1.11 (Vicon Motion Systems, Oxford, UK) was used to handle the transmission of the marker data.

### Design and procedure

Data for each participant was acquired in a single session. Upon arrival, participants were briefed on the study methods, and written informed consent was given by themselves or their legal guardian. With all systems attached, the marker model of the ball and the participant’s hand were measured using Vicon Nexus, as well as the minimal distance between the ball center and the fingertip of their throwing hand. This is represented by the average distance between the marker on the fingertip and the midpoint of the two pole markers on the ball, while the participant’s finger is resting on the ball’s surface for one second. Participants then performed a minimum of 10 warm-up throws to familiarize themselves with the setup. Afterward, each participant completed 500 basketball free throws with the individually preferred throwing hand. To avoid fatigue, participants were encouraged to take breaks between trials whenever necessary. In every second trial (occlusion condition), auditory information was withdrawn by playing white noise on their in-ear headphones. Additionally, their vision was occluded at the moment of ball release for 2.5 s, based on the online kinematic data. In the occlusion condition, participants were instructed to verbally predict the outcome of their throw by stating either ‘hit’ or ‘miss’ as soon as they reached a decision. This protocol was designed to quantify response time and minimize memory effects. Two experimenters caught the rebound, and noise-canceling acoustic foam mats were placed on the ground below the basket and the wall below the ring to avoid local and temporal feedback of the ball’s impact on the ground. Normal feedback was available in the other half of the trials, and no prediction had to be made. This approach was used to ensure a regular recalibration of the forward model and the actual sensory feedback [5]. An experimenter manually recorded the outcome of every throw (hit/miss) in the experimental software via a button press to compare the participants’ predictions with the actual results.

### Data analysis

When asked to predict the outcome of basketball free throw outcomes, participants are faced with a discrimination task of successful and unsuccessful trials. Hence, previous studies often used signal detection theory (SDT) to analyze the separation between the signal of a hit and the alternative signal of a miss. Such settings focused on the occurrence of the participants’ response behavior, resulting in the sensitivity parameter *d’*. This value depends on the amount of correctly and incorrectly stated hits (hit rate and false-alarm rate) and quantifies the distance between the signal distribution means, concerning their standard deviation. To describe the sensitivity to discriminate the signal of hit and miss trials, the parameter *d’* was calculated individually for every participant. Further, the general tendency to answer hit or miss, independent of the actual result, was calculated as the response bias *c*. The value *c* is quantified as the negative mean of the z-transformed hit rate and false alarm rate and represents the distance from a neutral point to the response criterion [24,25].

While SDT puts the occurrence and the distribution of signal and noise into perspective, it is hard to envision the consequences for the individual performing the decision. The present study aims to add a more tangible picture of decision-making, which does not rely on distribution assumptions. In this context, an approach based on Maurer and colleagues [12] was used, who developed a method to quantify the prediction accuracy in relation to a performance-dependent chance level, which describes the participants’ capabilities to predict their trial outcomes. The approach considers an individual chance rate for correct answers and quantifies the relative proportion of correctly predicted throws above this chance rate. Following this rationale, individual prediction accuracies (*%Acc*_*Pred*_) were calculated, which describe the participants’ capabilities to predict their free throw outcomes. Based on the formula for unconditional probability, the chance rate of correct answers (%C_Chance_) was calculated for each participant, assuming that participants’ answers were selected by chance (1). The rates of actual hits (Act_Hit_) and misses (Act_Miss_), as well as the rate of verbally predicted hits (Verb_Hit_) and misses (Verb_Miss_), are used in the calculation, which accounts for individual biases. The percentage of correct predictions (%C_Pred_) above the individual chance level (%C_Pred_-%C_Chance_) is normalized to the possible range of correct answers above the chance level (100-%C_Chance_) (2). Assuming that the verbal prediction is based on an internal model, this is a conditional probability, and %C_Pred_ should deviate from %C_Chance_.


$$\%C_{Chance}=\left({Act}_{Hit}*{Verb}_{Hit}+{Act}_{Miss}*{Verb}_{Miss}\right)*100$$ (1)

$$\%{Acc}_{Pred}=\frac{\left(\%C_{Pred}-\%C_{Chance}\right)}{\left(100-\%C_{Chance}\right)}$$ (2)

The measure used for the *%Acc*_*Pred*_ describes the extent to which the exploitable range above the chance level was utilized. If a person correctly predicts 5% more throws than the individual chance level, this leads to a higher value for a chance level of 75% (namely 20% *%Acc*_*Pred*_) than for a person with a chance level of 50% (namely 10% *%Acc*_*Pred*_).

Participants’ responses were captured by a microphone and analyzed post hoc. Sound data was filtered using a second-order Butterworth filter with a frequency band of [500 Hz, 4000 Hz]. Afterward, the voltage data were offset-corrected, rectified, and smoothed by a moving average with a window size of 25 ms. The highest voltage within the preprocessed data was set as the maximal amplitude. The closest data point before the maximum, smaller than 5% of the maximum, was used to identify the onset of the verbalized result relative to ball release. To account for possible individual differences in participants’ speech characteristics or the position of the microphone, the median latency and amplitude of each participant were used for normalization.

### Statistical analysis

MATLAB Version 2021a was used for data processing, and JASP Version 0.18.3 (University of Amsterdam, Amsterdam, Netherlands) was used for statistical analysis. For all tests, the alpha level was set to *p* < 0.05. Effect sizes were determined as *ƞ*^*2*^ and *ƞ*^*2*^_*part*_ [26]. In addition to frequentist statistics, a Bayesian inference approach was used to get further information about the weight of evidence favoring the null or alternative hypothesis. The Bayes Factor (BF) quantifies the ratio by which the observed data changes the prior to posterior odds. A BF of 1–3 was categorized as anecdotal evidence, 3–10 as sufficient, 10–30 as strong, 30–100 as very strong, and above 100 as extreme evidence [27]. Uninformed priors were used for the statistical analyses due to a lack of information about the parameter distribution in predictions of complex whole-body movements.

To check for a performance loss due to the verbal prediction differences, the hit rate between trials with and without prediction was tested using a one-tailed two-sample dependent *t*-test. To examine possible warm-up or fatigue effects in the throwing performance, the hit rate of the first and last 20% of the trials (100 trials each) was calculated and compared using a two-tailed one-sample student’s *t*-test.

The sensitivity to discriminate hits and misses, as well as the response bias, were analyzed by one-tailed one-sample student’s *t-*tests on *d’* and *c* values. A one-tailed one-sample student’s *t*-test was used to test whether the *%Acc*_*Pred*_ of participants was above individual chance level. To examine changes in the prediction performance throughout the experiment, the *%Acc*_*Pred*_ was calculated over blocks of 20% (50 prediction trials each), and the first and last blocks were compared using a two-tailed one-sample student’s *t*-test. Pearson correlations were computed to examine the relationship between hit rate and individual *c* values, as well as between hit rate and *%Acc*_*Pred*_, and hit rate and *d*’ value. Furthermore, a Pearson correlation was conducted to examine the relationship between participants’ *%Acc*_*Pred*_ and individual *d’* values.

Speech data (amplitude and latency) was grouped into four result categories (actual hit/miss and verbalized hit/miss). Two 2 (actual result: hit vs. miss) x 2 (verbalized result: hit vs. miss) repeated measures analysis of variance were applied to compare the relative latency and amplitude of the verbalized throwing results between the four categories. Two Pearson correlations were used to explore the relationship between the difference in speech data (amplitude and latency) of verbal results (hit – miss) and the individual hit bias.

Single trials were excluded from all analyses if an executed throw or a verbalized report was flagged invalid during the data acquisition (e.g., because markers became detached during movement execution or because of technical issues in online marker tracking). Further, trials were omitted if participants did not answer or could not decide on one option and changed their answer within a trial. For the analysis of the speech characteristics, shots were excluded if the response was valid, but either the onset latency or the maximal amplitude could not be detected properly due to technical issues. Data from one participant could not be used because no speech data was captured due to technical issues. This participant’s verbal prediction responses were captured manually. In total, 0.82% of the trials had to be excluded from all analyses and 3.44% from the speech analysis.

## Results

### Throwing performance

The average hit rate of all participants was 63.41% (SD = 13.32%). The comparison of hit rates between trials with and without verbal prediction did not show any significant difference [*t*_18_ = 0.61, *p = *0.72, *ƞ*^*2*^ < 0.01, *BF*_*01*_ = 6.24]. The comparison between the first and last 20% of the trials revealed a significantly higher hit rate at the end of the session [*t*_18_ = −3.31, *p* < 0.01, *ƞ*^*2*^ = 0.13, *BF*_*10*_ = 11.55].

### Prediction performance

**Fig 1:**
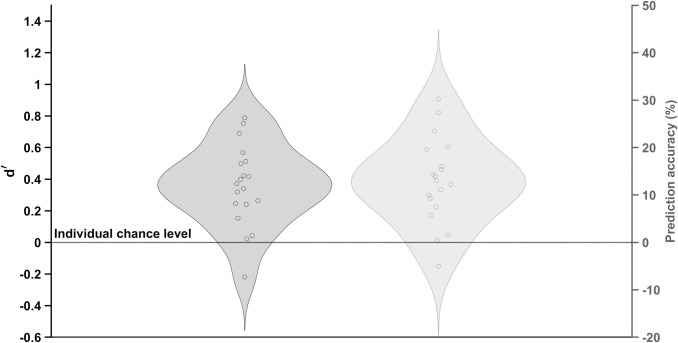
Distribution of d’ and prediction accuracy values.

With an average value of 0.36 (SD = 0.26), the *d*’ values deviated highly significantly from zero [*t*_18_ = 6.14, *p* < 0.01, *ƞ*^*2*^ = 0.33, *BF*_*10*_ = 5112.56], indicating correct discriminations (Fig 1). The decision criterion *c* reached a mean value of −0.32 (SD = 0.36) and a highly significant difference from zero [*t*_18_ = −3.86, *p* < 0.01, *ƞ*^*2*^ = 0.16, *BF*_*10*_ = 66.02] with a tendency to answer ‘hit’. Moreover, it was highly significantly correlated with the participants’ hit rate [*r*_18_ = −0.64, *ƞ*^*2*^ = 0.40, *p* < 0.01, *BF*_*10*_ = 15.24], indicating that athletes with higher throwing performance have a stronger bias for hit answers.

Furthermore, the average *%Acc*_*Pred*_ of 12.97 (SD = 8.98), indicating correct predictions above their individual chance level, showed a highly significant difference from zero [*t*_18_ = 6.30, *p* < 0.01, *ƞ*^*2*^ = 0.34, *BF*_*10*_ = 6809.27] (Fig 1). However, no correlation was found between participants’ hit rate and their *%Acc*_*Pred*_ [*r*_18_ = −0.16, *ƞ*^*2*^ = 0.03, *p* = 0.52, *BF*_*01*_ = 2.89] or their *d’* value [*r*_18_ = −0.11, *ƞ*^*2*^ = 0.01, *p* = 0.66, *BF*_*01*_ = 3.22]. A highly significant correlation was found between *%Acc*_*Pred*_ and *d’* values [*r*_17_ = 0.99, *ƞ*^*2*^ = 0.98, *p* < 0.01, *BF*_*10*_ = 6.45 x 10^11^]. The analysis of *%Acc*_*Pred*_ between the first and last blocks did not reveal any significant difference [*t*_18_ = 0.67, *p* = 0.51, *ƞ*^*2*^ = 0.01, *BF*_*01*_ = 3.52].

### Speech characteristics

**Fig 2:**
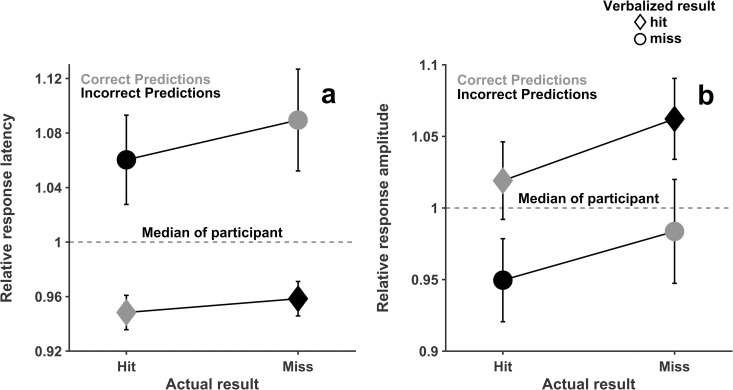
Analysis of speech characteristics based on result pattern. (a) Relative latency based on the result pattern. (b) Relative amplitude based on the result pattern.

In the 2x2 repeated measures analyses of variance, the relative latency for verbalized misses was significantly higher than for verbalized hits [*F*_1,17_ = 13.62, *p* < 0.01, *ƞ*^*2*^_*part*_ = 0.44, *BF*_*10*_ = 18.38]. No further effects regarding the variable response latency were significant (actual result: [*F*_1,17_ = 1.25, *p* = 0.28, *ƞ*^*2*^_*part*_ = 0.07, *BF*_*01*_ = 2.33], verbalized results x actual results: [*F*_1,17_ < 0.01, *p* > 0.99, *ƞ*^*2*^_*part*_ < 0.01, *BF*_*01*_ = 0.41]) (Fig 2a).

A significant difference in the factor verbalized result could also be shown for the relative response amplitude, with higher amplitudes for verbalized hits than misses [*F*_1,17_ = 5.54, *p* = 0.03, *ƞ*^*2*^_*part*_ = 0.25, *BF*_*10*_ = 1.72]. No other comparisons were significant for the relative response amplitude (actual result: [*F*_1,17_ = 0.81, *p* = 0.38, *ƞ*^*2*^_*part*_ = 0.05, *BF*_*01*_ = 2.27], verbalized results x actual results: [*F*_1,17_ < 0.01, *p* = 0.89, *ƞ*^*2*^_*part*_ < 0.01, *BF*_*01*_ = 3.94]) (Fig 2b).

**Fig 3:**
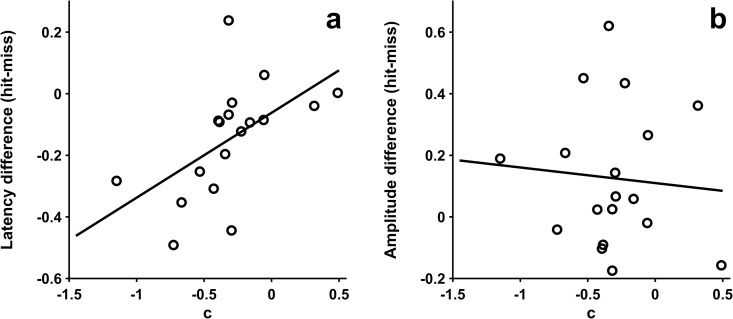
Correlation between hit bias c and speech characteristics. (a) Correlation of response bias c and response latency. (b) Correlation of response bias c and response amplitude.

The difference in response latency between the verbal responses (hit – miss) showed a significant correlation with participants’ hit bias *c* [*r*_16_ = 0.55, *ƞ*^*2*^ = 0.30, *p* = 0.02, *BF*_*10*_ = 3.99] (Fig 3a), indicating faster ‘hit’ answers compared to miss answers with a larger bias of answering ‘hit’. The difference in response amplitude did not correlate with the hit bias [*r*_16_ = −0.08, *ƞ*^*2*^ = 0.01, *p* = 0.75, *BF*_*01*_ = 3.27] (Fig 3b).

## Discussion

This study aimed to explore whether basketball players can verbally predict their own free throw outcomes above chance, examining the role of performance and potential biases in prediction accuracy in motor experts. Therefore, athletes were challenged to make predictions under uncertainty without visual or auditory feedback after ball release. It was expected that a proficient forward model, which experts are assumed to have, complemented by the ability to access this internal representation, would facilitate correct predictions above chance level.

### Throwing performance

Participants had an average basketball experience of 11 years and performed their free throws with an average hit rate of 63%. Notably, this experience level, based on active years of playing basketball, was in a similar range as in earlier studies on predictions with basketball players [10,28] or even exceeding it [9]. However, in contrast, comparable studies reported lower throwing performances, with hit rates of only 41% and 50%, respectively [9,14]. This discrepancy in performance may be explained by methodological differences, for instance, the absence of feedback during the experimental trials [29] or a negative-attention effect of the preparation of the verbal response (according to the theory of reinvestment [30]). Further, minimizing the influence of attached equipment may be critical when assessing ecologically valid performance [14]. To better contextualize and interpret laboratory-based hit rates, having access to players’ performance data under natural conditions would be beneficial, thereby increasing the validity of cross-study comparisons. Nevertheless, in the present study, an increase in the hit rate over the course of the experimental session was even observed, indicating a familiarization with the laboratory situation. Moreover, while prior studies [12,14] observed a decrease in throwing performance when verbal outcome predictions were required, particularly among less-skilled players, no such prediction-related performance cost was found in the present study. This suggests that the observed participants, as a group, may have possessed a higher level of expertise or were better able to integrate the dual demands of performance and verbal prediction. It is important to note that the change in throwing performance is not accompanied by a change in prediction performance, indicating robust internal models.

### Prediction performance

On average, the participants in the present study reached a *d*’ value of 0.36. The significant difference from zero reveals the participants’ ability to discriminate between successful and unsuccessful trials, which indicates that experienced basketball players have a good internal representation of their basketball free throws. The values reach a similar range as in other studies with recreational basketball players [14]. More experienced participants showed even higher detection values than observed in this study [9,14]. In both studies, though, visual feedback was manually withdrawn by an experimenter, which may have delayed the occlusion and led to an unintentional provision of information about the ball flight after release. Nonetheless, the overall trend strengthens the hypothesis that motor experts can predict the outcome of a highly trained movement, such as the free throw.

In addition to the SDT measure *d’*, prediction performance was determined by an individual prediction accuracy measure, showing the extent to which a participant’s predictions exceed their personalized chance level. The participants showed an average *%Acc*_*Pred*_ of 12.97%, which was significantly above their individual chance levels. This captures the degree to which a participant effectively utilizes their predictive abilities. The values achieved were even higher than in a previously studied semi-virtual throwing task [12]. This can be explained by having greater experience with the specific task and therefore a more pronounced internal model. The basketball players in the present study had years of experience, while participants performing the semi-virtual task built up their experience in just 1000 trials.

As *d’* and *%Acc*_*Pred*_ values were not very high, participants retained a limited ability to predict outcomes above their chance level. However, their performance has been constrained by the lack of sensory feedback, by depriving visual and auditory information after ball release. This means that input to the prediction and discrimination performance could only be based on efferent and kinesthetic information. Hence, results indicate that participants had access to the output of their forward model while throwing and were able to rely on this to verbally predict the outcome of their movement after ball release.

Furthermore, a high correlation between *%Acc*_*Pred*_ and *d*’ values was found. Assuming experienced basketball players can access their sensorimotor online predictions, a strong relationship between the separation of different signals and prediction on a performance level seems conclusive. However, no relationship could be found between participants’ throwing performance and outcome prediction (*d’* and *%Acc*_*Pred*_). This result is similar to Abreu and colleagues [28] but contrasts with that of Maglott and colleagues [14], who reported an increased prediction ability with higher skill levels of different experience groups. A positive relationship between performance and access to internal models could be expected when comparing a spectrum of different experience levels [11]. A comparison of basketball experts with novices or athletes from different sports, similar to earlier studies [10,14], could expand the knowledge of how internal models develop with expertise and are used to predict the outcome of motor actions.

Additionally, signal detection theory offers the possibility to quantify a potential response bias [25]. The average *c* value of −0.32 revealed that participants tended to prefer to answer ‘hit’. This direction is in line with earlier studies, which found a similar hit bias when basketball players predicted their free throws [9,14]. Across different sports disciplines, especially in basketball, a promotion focus could be identified, characterized by prioritizing growth and winning instead of safety and losing [19,31]. These findings align with the positive correlation between *c* and the overall hit rate. More advanced players seem to accept a higher false alarm rate to increase their amount of correctly predicted hit trials. The certainty of participants’ answers could be further investigated by additional measures of metacognitive parameters [32]. This would provide more insight into whether athletes answer ‘hit’ more often when in doubt, or when convinced of the positive outcome of their shot.

### Speech characteristics

The latency and amplitude of the verbal responses differed as a function of the verbalized outcome. When participants answered ‘hit’, they took less time to respond and answered louder relative to a ‘miss’ answer, regardless of the actual outcome. This result pattern matches the outcome of Maurer and colleagues [12], where a virtual throwing task and novice participants were observed. There, speech characteristics of outcome predictions showed a later and quieter response articulation when participants verbally predicted an unsuccessful outcome. This pattern could be found especially in incorrect predictions of successful trials. In contrast, the examination of latency and amplitude in the current study did not reveal any interaction effects between the verbalized responses and the actual trial outcomes. However, that the articulation of successful throws was faster and louder, independent of the actual outcome, is in line with the idea that expert outcome predictions are influenced by an overall hit bias (higher with increasing hit rate), requiring a longer time to switch the inner belief to a miss. Additionally, the latency difference between the two responses (hit and miss) was larger in participants who showed a larger hit bias. Thus, it seems that the latency difference is driven by the hit bias, and the tendency to prefer answering ‘hit’ is manifested in the response articulation and speeds up reporting a successful free throw. This matches the idea of a prediction process, in which increased hit-miss discrepancies are assumed to result from a more pronounced response bias. Without any bias, the reaction to hit trials would take just as much time as for missed trials. A small prior to answer ‘hit’ would lead to slightly faster responses for successful trials and slightly more time to accept unsuccessful outcomes. Therefore, only small differences would be noticeable. As the bias becomes more prominent, ‘hit’ answers become easier and faster, while it takes even more evidence and time to change the belief to a ‘miss’. This combination results in a higher deviation between the two response possibilities due to an increased bias. Alternatively, a contrast in speech production between the two response options, ‘hit’ and ‘miss’, could explain the finding of hit-miss differences. As both words are of equal length and the consonant-vowel sequence is similar, this is not considered to be very likely. However, future research could study the influence of different response options, including vocal and non-vocal responses (e.g., push buttons). Moreover, interpreting the verbal prediction as a two-choice decision task, the response time distribution and the trade-off between accuracy and speed as influencing factors, could be studied using a diffusion drift model [18].

## Conclusion

In the context of motor control, people rely on different internal models to gather information about the commands sent to the effectors and the predicted sensory consequences based on the current state of the body and the environment. When experienced basketball players are asked to verbally predict the final outcome of their actions, e.g., a basketball free throw, they can report the result and exceed their individual chance level. Therefore, they must rely on evidence based on motor commands as well as additionally integrated information about the flow of events during movement execution. The time at which they are confident enough to make their decision is quantified as the response latency. The speed as well as the amplitude of their response is highly influenced by their bias to report successful trials, leading to faster and louder ‘hit’ articulations.
